# Adaptation to second order stimulus features by electrosensory neurons causes ambiguity

**DOI:** 10.1038/srep28716

**Published:** 2016-06-28

**Authors:** Zhubo D. Zhang, Maurice J. Chacron

**Affiliations:** 1 Department of Physiology, McGill University, Montreal, QC, Canada

## Abstract

Understanding the coding strategies used to process sensory input remains a central problem in neuroscience. Growing evidence suggests that sensory systems process natural stimuli efficiently by ensuring a close match between neural tuning and stimulus statistics through adaptation. However, adaptation causes ambiguity as the same response can be elicited by different stimuli. The mechanisms by which the brain resolves ambiguity remain poorly understood. Here we investigated adaptation in electrosensory pyramidal neurons within different parallel maps in the weakly electric fish *Apteronotus leptorhynchus*. In response to step increases in stimulus variance, we found that pyramidal neurons within the lateral segment (LS) displayed strong scale invariant adaptation whereas those within the centromedial segment (CMS) instead displayed weaker degrees of scale invariant adaptation. Signal detection analysis revealed that strong adaptation in LS neurons significantly reduced stimulus discriminability. In contrast, weaker adaptation displayed by CMS neurons led to significantly lesser impairment of discriminability. Thus, while LS neurons display adaptation that is matched to natural scene statistics, thereby optimizing information transmission, CMS neurons instead display weaker adaptation and would instead provide information about the context in which these statistics occur. We propose that such a scheme is necessary for decoding by higher brain structures.

Growing evidence suggests that natural scene statistics constrain neural coding strategies^1^,^2^,^3^. Specifically, it is thought that information is efficiently coded by ensuring that neurons can adapt their tuning properties to stimulus statistics^1^,^3^. However, sensory adaptation can also cause ambiguity as the same neural response can then be elicited by different stimuli^1^,^4^,^5^. The mechanisms by which such ambiguity is resolved in the brain remain poorly understood.

Weakly electric fish sense amplitude modulations (AMs) of a self-generated electric organ discharge (EOD) through an array of electroreceptors scattered on their skin surface^6^,^7^. Afferent fibers trifurcate and make synaptic contact with pyramidal neurons within three parallel somatotopic maps of the body surface (centromedial: CMS, centrolateral: CLS, and lateral: LS) in the hindbrain electrosensory lateral line lobe (ELL) (see ref. 8 for review). Natural electrosensory stimuli display rich spatiotemporal characteristics. In particular, when two conspecifics come into close contact, interactions between their EODs give rise to a sinusoidal AM (first order) with frequency equal to the difference between the individual EOD frequencies and whose amplitude (second order) varies depending on the relative distance and orientation between both fish^9^,^10^,^11^ (see ref. 12 for review). While much is known about how ELL pyramidal neurons respond to first order features of electrosensory stimuli (for review see refs 6,8,13), considerably less is known about how they respond to second order features^14^,^15^,^16^,^17^,^18^,^19^,^20^. In particular, the adaptation properties of pyramidal neurons across different segments to the second order features of electrosensory stimuli and their consequences on coding are unknown.

Here we investigated how pyramidal neurons within the CMS and LS maps respond to step changes in second order electrosensory stimuli. We found stronger adaptation in LS than CMS neurons. The time course of the adaptation in both segments was scale invariant and could be well-fit by a power law with exponent α. Thus, the adaptation was matched to the stimulus statistics as the apparent time scale varied with step duration as assessed by instead fitting an exponential to the data. We then used signal detection theory^21^ to assess the ability of an ideal observer to discriminate between the firing rate distribution immediately before step onset and the distributions immediately after (i.e. before adaptation) and immediately before step offset (i.e. after adaptation). Our results show that adaptation can significantly reduce discriminability as quantified by the area under the receiver operating characteristic (ROC) curve (AuC) and suggest that ambiguity is resolved by processing inputs from CMS and LS pyramidal neurons in parallel.

## Results

### ELL pyramidal neurons display spike frequency adaptation in response to envelope steps

**Figure 1:**
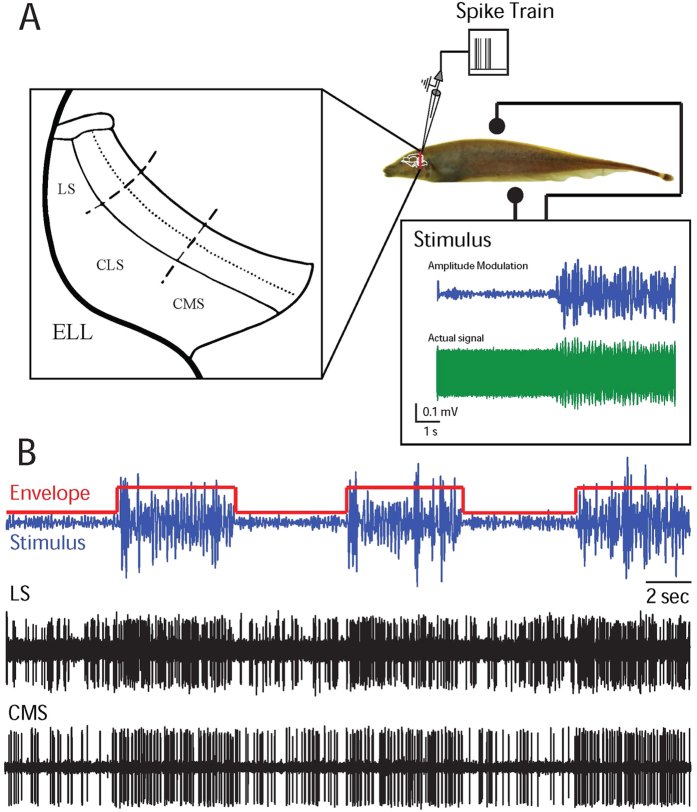
ELL pyramidal neurons across different maps respond differentially to envelope steps. **(A)** Schematic of the experimental setup. Amplitude modulations of the animal’s own electric field are delivered via two electrodes on the side while ELL pyramidal neurons within the LS and CMS maps are recorded from. (**B)** Stimulus waveform (blue) and its envelope (red) (top) as well as recordings from example LS (middle) and CMS (bottom) pyramidal neurons.

We obtained extracellular recordings from n = 45 LS and n = 32 CMS ELL pyramidal neurons *in vivo* (Fig. 1A). Stimuli consisted of amplitude modulations of the animal’s own EOD whose time course mimicked the stimulation caused by interference between the EODs of two conspecifics and whose variance switched periodically between a low and a high value (Fig. 1A,B). It is important to note that the EOD is a carrier waveform and that the relevant stimulus here is the EOD AM (Fig. 1A,B, blue). We then quantified adaptation in ELL pyramidal neuron responses to step changes in stimulus amplitude (Fig. 1B, red). For this reason, we will refer to the EOD AM as first order and to the envelope as second order, respectively. We note that these correspond to the second and third order features of the full signal received by the animal (Fig. 1A, green), respectively. Figure 1B shows the extracellularly recorded responses from example LS (middle) and CMS (bottom) pyramidal neurons. Both cells responded to the envelope step onset through an increase in firing rate whose subsequent decay was more pronounced in the LS neurons (Fig. 1B).

**Figure 2:**
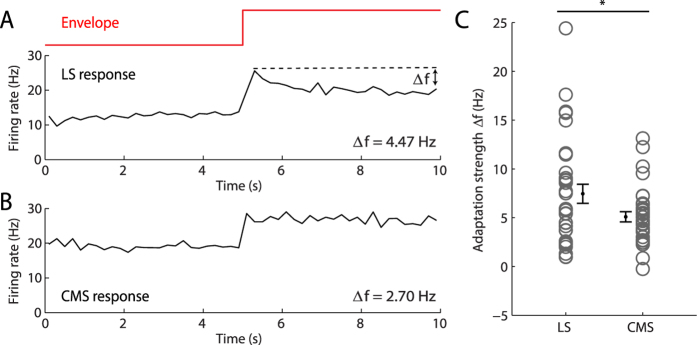
LS and CMS ELL pyramidal neurons display differential degrees of adaptation to envelope steps. **(A)** Peri-stimulus time histogram (PSTH) from an example LS neuron (black) in response to the envelope step (red) with binwidth = 500 msec. We computed the strength of adaptation as the difference between the firing rate at step onset and offset Δf. (**B)** PSTH response from an example CMS neuron. Note the lesser degree of adaptation. (**C)** LS pyramidal neurons display significantly larger adaptation strengths (left, n = 45) than CMS (right, n = 32) pyramidal neurons (p = 0.0377, one-way ANOVA). The gray open circles show the adaptation strength of each neuron while population-averages with SEM are shown in black.

We characterized spike frequency adaptation by averaging neural responses across step onsets and plotting the time dependent firing rate as a peri-stimulus time histogram (PSTH). Plotting the PSTH for an example LS pyramidal cell revealed that the cell responded to the step onset by a sharp increase in firing rate followed by a slower decay that is characteristic of spike frequency adaptation (Fig. 2A). In contrast, an example CMS pyramidal cell responded to the step onset by a similar sharp increase in firing rate that did not decay as much (Fig. 2B). We quantified the tendency of cells to display spike frequency adaptation in response to envelope steps by plotting the difference between the maximum firing rate and the firing rate just before step offset (i.e. the adaptation strength). Comparing datasets from LS and CMS neurons revealed that the former tended to display significantly more adaptation than the latter (Fig. 2C). Interestingly, no significant difference in adaptation strength was observed when comparing values for ON and OFF-type pyramidal neurons in either LS or CMS (p > 0.1 in each case). The implications of this result are discussed below.

### Adaptation to envelopes in pyramidal neurons is scale invariant

What is the time course of adaptation in ELL pyramidal neurons? To answer this important question, we fitted both exponential and power law models to our data in response to step changes in envelope at frequencies between 0.05 and 16 Hz thereby varying the step duration. If adaptation to envelopes displays a characteristic timescale, then we would expect that PSTH responses to step onset with different duration will all be well-fit by an exponential curve with the same time constant. If, in contrast, adaptation to envelopes were scale invariant, then we would expect that PSTH responses to step onset with different duration would all be well-fit by a power law curve with the same exponent. The apparent decay time constant of adaptation as quantified by fitting an exponential is then proportional to the envelope duration^4^,^22^.

**Figure 3:**
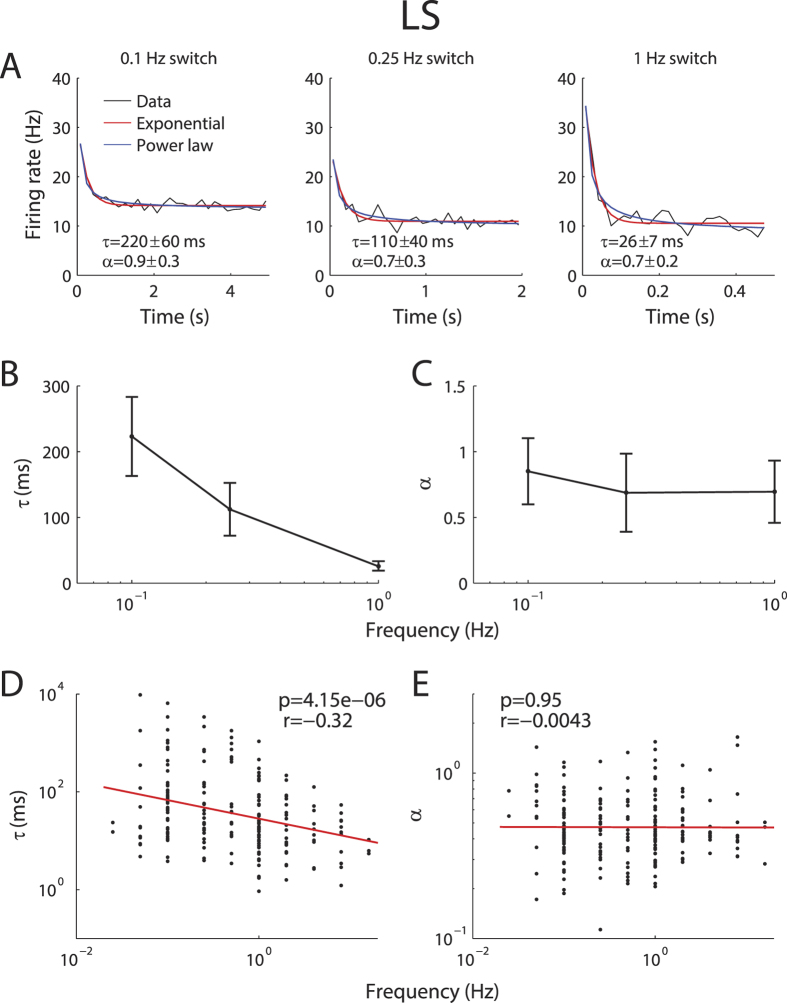
LS pyramidal neurons display power law adaptation to envelope steps. **(A)** PSTH response (black) from an example LS neuron to envelope steps switching at rates of 0.1 Hz (left), 0.25 Hz (center), and 1 Hz (right). Also shown are exponential (red) and power law (blue) fits with time constants and exponents, respectively. Note that the power law exponents obtained for each rate were similar whereas large differences were observed for the time constants. (**B)** Exponential time constants for the fits in (**A**) with error bars as 95% confidence intervals. (**C)** Power-law exponents for the fits in (**A**), with 95% confidence intervals. (**D)** Best-fit exponential time constants from individual neurons as a function of switching frequency. We observed a significantly negative correlation (R = −0.32, p ≪ 0.01). (**E)** Best-fit power law exponent from individual neurons as a function of switching frequency. Both quantities were not significantly correlated (R = −0.0043, p = 0.95).

Our results show that LS pyramidal neuronal adaptation to step changes in envelope with different durations were all well-fit by power laws with similar exponents (Fig. 3A, compare black and blue). In contrast, while each curve could also be well-fit by an exponential, the time constant decreased when the step duration decreased (Fig. 3A, compare black and red). Similar results were seen across our dataset as the population-averaged exponential time constant decreased as a function of step frequency (Fig. 3B) while the power law exponent did not (Fig. 3C). As a result, the time constant of the best exponential fit and switching frequency were significantly negatively correlated (*r* = −0.317, *p* < 10^−5^) (Fig. 3D). In contrast, the exponent of the best power law fit was constant as a function of switching frequency (*r* = −0.004, *p* > 0.95) (Fig. 3E). We conclude that LS pyramidal neurons display power law adaptation in response to step changes in envelope.

**Figure 4:**
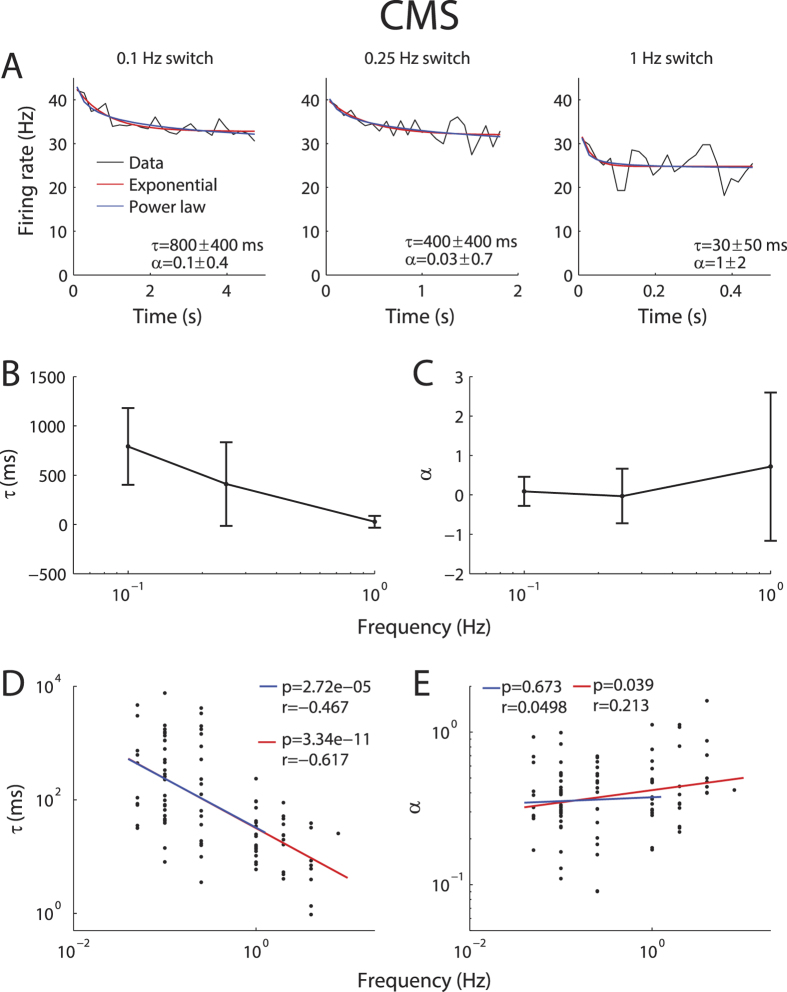
CMS pyramidal neurons display power law adaptation to envelope steps. **(A)** PSTH response (black) from an example CMS neuron to envelope steps switching at rates of 0.1 Hz (left), 0.25 Hz (center), and 1 Hz (right). Also shown are exponential (red) and power law (blue) fits with time constants and exponents, respectively. (**B)** Exponential time constants for the fits in (**A)** with error bars as 95% confidence intervals. (**C)** Power-law exponents for the fits in (**A**) with 95% confidence intervals. (**D)** Best-fit exponential time constants from individual neurons as a function of switching frequency. We observed a strong and significantly negative correlation for frequencies up to 1 Hz (blue, R = −0.47, p ≪ 0.01) and 8 Hz (red, R = −0.62, p ≪ 0.01). (**E)** Best-fit power law exponent from individual neurons as a function of switching frequency. Both quantities were not significantly correlated (R = −0.0043, p = 0.95) for frequencies up to 1 Hz (blue). When including higher frequencies up to 8 Hz, we observed a weak but significantly positive correlation (red, R = 0.21, p = 0.04).

While our results show that CMS pyramidal neurons displayed significantly less adaptation than their LS counterparts, it was possible in general to characterize the timecourse of adaptation in these cells. Overall, qualitatively similar results were seen in that responses to different step durations were all well fit by a power law with similar exponents (Fig. 4A, compare black and blue). While each curve could be well-fit by an exponential, the time constant of the best exponential fit strongly varied with stimulus duration (Fig. 4A, compare black and red). Similar results were seen across our CMS dataset as the population-averaged exponential time constant decreased as a function of step frequency (Fig. 4B) while the power law exponent did not (Fig. 4C). As a result, the exponential time constant was strongly and significantly negatively correlated with switching frequency (*r* = −0.6, *p* ≪ 0.001) (Fig. 4D). In contrast, the power law exponent was not significantly correlated with switching frequency for up to 1 Hz (*r* = 0.0498, *p* > 0.5) and only a weak positive correlation was observed when taking into account all frequencies (*r* = 0.213, *p* > 0.5) (Fig. 4E).

### Adaptation to envelope steps by ELL pyramidal neurons causes ambiguity

We next examined the functional role of adaptation to envelopes in ELL pyramidal neurons. While previous studies have made it clear that adaptation can greatly enhance information processing by optimizing neural responses to natural stimuli^1^, it is also clear that adaptation can also cause ambiguity in the neural response^1^,^4^. To quantify ambiguity resulting from adaptation, we used signal detection theory to quantify the performance of an ideal observer as discriminating between the neural response preceding the step onset and the neural response at various times following the step onset^21^.

**Figure 5:**
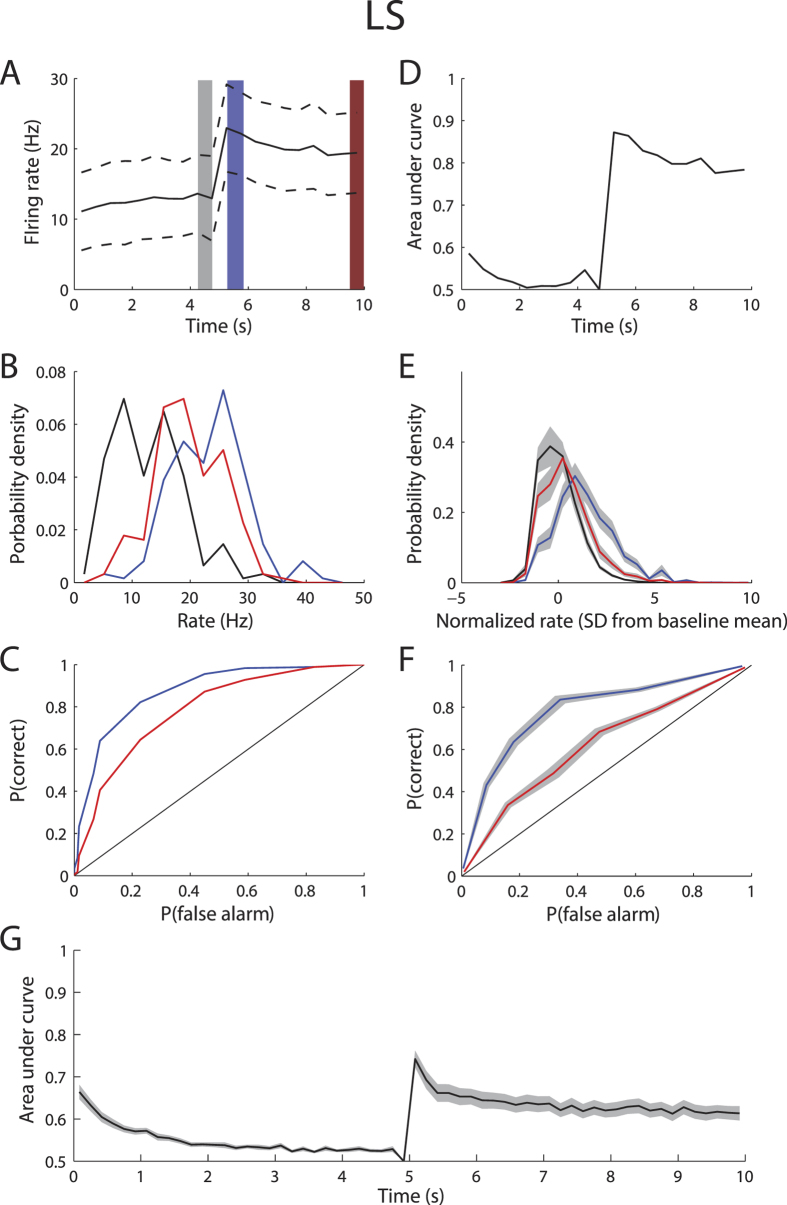
Adaptation to envelope steps causes ambiguity in LS pyramidal neurons. **(A)** PSTH response from the same example LS neuron shown in Fig. 2A (black) and standard deviation (dashed black lines). We compared the firing rate distributions immediately after step onset (blue) and after adaptation before step offset (red) to that immediately before step onset (gray). (**B)** Firing rate probability densities immediately before step onset (black), immediately after step onset (blue), and immediately before step offset (red). (**C)** ROC curves from chance (black), immediately after step onset (blue), and after adaptation (red). (**D)** Area under the ROC curve (AuC) as a function of time with reference being immediately before step onset. (**E)** Population-averaged firing rate probability densities immediately before step onset (black), immediately after step onset (blue), and immediately before step offset (red). The firing rate was normalized by first subtracting the mean baseline and dividing by the standard deviation. (**F)** Population-averaged ROC curves from chance (black), immediately after step onset (blue), and after adaptation (red). (**G)** Population-averaged AuC as a function of time. The gray bands show 1 SEM.

Results from an example LS neuron shows that spike frequency adaptation impairs discriminability (Fig. 5A). Indeed, there was less overlap between the firing rate distribution immediately before and after the step onset, before adaptation, than between the firing rate distributions immediately before the step onset and offset, after adaptation, (Fig. 5B) as reflected in the ROC curves (Fig. 5C). We quantified discriminability by computing the area under the curve (AuC) for ROC curves and plotting this quantity as a function of time. Our results show that the AuC increased markedly after step onset and showed a pronounced decay. Similar results were seen across our LS dataset in terms of overlap between distributions (Fig. 5E), ROCs (Fig. 5F), and the timecourse of the AuC (Fig. 5G). We note that the AuC remained significantly above chance level even 5 sec after step onset indicating that some discriminability is achievable at the single neuron level.

**Figure 6:**
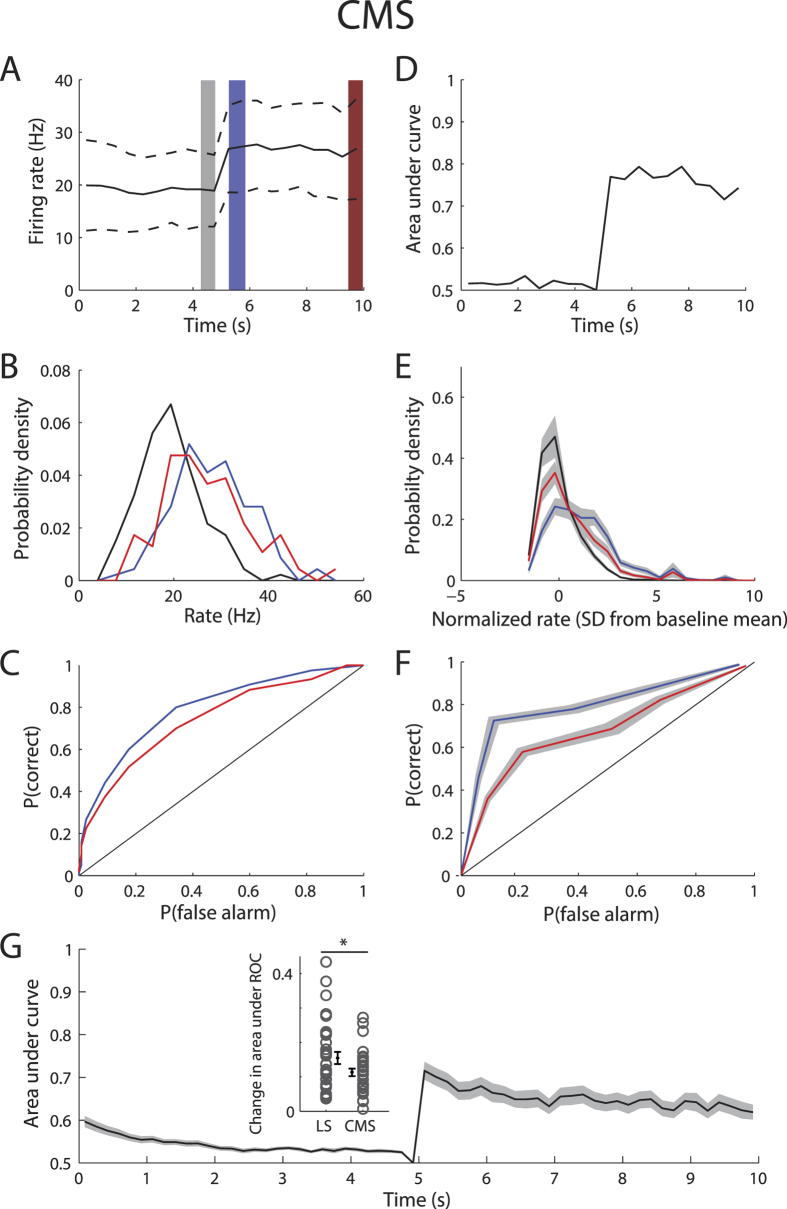
Adaptation to envelope steps causes less ambiguity in CMS pyramidal neurons. **(A)** PSTH response from the same example CMS neuron shown in Fig. 2B (black) and standard deviation (dashed black lines). We compared the firing rate distributions immediately after step onset (blue) and after adaptation before step offset (red) to that immediately before step onset (gray). (**B)** Firing rate probability densities immediately before step onset (black), immediately after step onset (blue), and immediately before step offset (red). (**C)** ROC curves from chance (black), immediately after step onset (blue), and after adaptation (red). (**D)** Area under the ROC curve (AuC) as a function of time with reference being immediately before step onset. (**E)** Population-averaged firing rate probability densities immediately before step onset (black), immediately after step onset (blue), and immediately before step offset (red). (**F)** Population-averaged ROC curves from chance (black), immediately after step onset (blue), and after adaptation (red). (**G)** Population-averaged AuC as a function of time. The gray bands show 1 SEM. Inset: The change in AuC was significantly greater for LS (left) than for CMS (right) neurons (p < 0.05, one-way ANOVA).

We next investigated whether the weaker degree of adaptation displayed by CMS pyramidal neurons (Fig. 6A) gave rise to greater discriminability. Our results show that overlap between the firing rate distributions immediately before and after the step onset was similar to that between those immediately before the step onset and offset (Fig. 6B) as reflected in the ROC curves (Fig. 6C). Consequently, the AuC remained more or less constant after the step onset (Fig. 6D). Qualitatively similar results were seen across our CMS dataset in terms of overlap between distributions (Fig. 6E), ROCs (Fig. 6F), and the timecourse of the AuC (Fig. 6G). Comparing results between CMS and LS revealed a significantly lesser change in AuC (i.e. the difference between the AuC at step onset and offset) for the former than for the latter (Fig. 6G, inset).

Thus, we conclude that adaptation can significantly reduce discriminability of responses to step changes in envelope. Discriminability impairment was greatest in LS and weakest in CMS. The implications of these results are discussed below.

## Discussion

### Summary of results

We investigated how pyramidal neurons within the ELL CMS and LS segments responded to second order step changes. We found that LS pyramidal neurons responded to such stimuli through an initial increase in firing rate followed by a slower decay whose timecourse was scale invariant as it was well fit by a power law independently of step duration. CMS pyramidal neurons also responded to the step onset with an increase in firing rate but displayed significantly less adaptation than their LS counterparts. We then used signal detection theory to quantify the ability of an ideal observer to discern the change in firing rate due to the step increase during the early and late phases of adaptation. Our results showed that adaptation in LS significantly reduced discriminability as the mean firing rate decreased closer to its value before step onset. In contrast, discriminability did not decrease as much for CMS neurons, consistent with their decreased tendency to display adaptation.

### Resolving ambiguity caused by adaptation

It is well known that adaptation in neural responses causes ambiguity as stimuli with different characteristics (e.g. intensity) can then give rise to the same neural response (e.g. firing rate)^1^,^4^,^5^. Our results have shown that the greater level of adaptation in LS neurons caused greater ambiguity than the lower levels seen in CMS neurons. How then does the brain resolve ambiguity caused by adaptation? One potential solution to this problem is to take advantage of the fact that the neural response immediately after the step can encode stimulus amplitude^23^. Our results show that this solution can also be implemented in the electrosensory system but requires that the stimulus after onset varies slowly, which is not typically seen under natural conditions^9^,^10^,^11^. Another solution is to use parallel coding such that information about first and second order stimulus attributes are encoded separately, either by different neural circuits or through different attributes of the neural response. Indeed, it was proposed that information about first and second order stimulus features could be encoded by firing rate and spike timing in within the same spiking response, respectively^4^. Our results however support the former hypothesis in that pyramidal neurons within different maps of the body surface can resolve ambiguity by displaying differential degrees of adaptation. Pyramidal neurons within all ELL segments project to the midbrain torus semicircularis (TS) (see ref. 8 for review). While the adaptation properties of TS neurons to second order attributes of electrosensory stimuli have not been systematically characterized to date, previous studies have shown that these neurons can respond selectively to different features of electrosensory stimuli such as movement^24^,^25^,^26^, natural communication stimuli^27^,^28^,^29^, and second order attributes^16^. In particular, the ambiguity caused by adaptation is most likely resolved in TS as different subsets of neurons each respond selectively to first and second-order features of electrosensory stimuli^16^. Further studies are however needed to understand how TS neurons integrate input from pyramidal neurons within all ELL segments.

### Mechanisms mediating adaptation by ELL pyramidal neurons to second order electrosensory stimuli

What are the mechanisms underlying the differential degree of adaptation to second order electrosensory stimuli observed in CMS and LS pyramidal neurons? Previous studies have shown that pyramidal neurons across the different ELL segments displayed differential expressions of SK channels^30^. Indeed, SK channel expression is lowest in CMS and highest in LS pyramidal neurons. Previous studies have also shown that SK channel expression was directly correlated with the differential degree of adaptation displayed by these cells to first order steps in current injection *in vitro*^31^,^32^. Here we have shown that this differential degree of SK expression also correlates with differential degrees of adaptation to second order steps in sensory stimulation *in vivo*. We note that only two SK channel subtypes are expressed in the ELL: SK1 and SK2, and both subtypes display the same pattern of graded expression amongst the three tuberous ELL segments^30^,^31^. However, while SK2 channels are located on the somata of ON-type pyramidal neurons only, SK1 channels are instead located on the apical dendrites of both ON- and OFF-type pyramidal neurons. Since we observed that both ON and OFF-type pyramidal neurons did not display major differences in terms of adaptation to second order stimuli, we hypothesize that it is SK1, rather than SK2, channels that likely mediate the differential adaptation properties of CMS and LS pyramidal neurons. As mentioned below, SK2 channels are more likely involved in the processing of AM stimuli. Importantly, pyramidal neurons receive large amounts of feedback on their apical dendrites^33^,^34^, which can strongly alter responses to sensory input^35^,^36^,^37^,^38^,^39^,^40^,^41^,^42^. It is thus likely that the differential adaptation properties of CMS and LS pyramidal neurons are due to differential integration of feedback input that is mediated by SK1 channels. Further studies are however needed to test this hypothesis.

Previous studies have shown that neuromodulators can strongly influence ELL pyramidal neuron tuning to sensory input^43^,^44^,^45^ (see refs 46,47 for review). In particular, serotonin increases ELL pyramidal neuron responses to stimuli associated with same sex conspecifics^44^ by inhibiting SK channels^43^. As mentioned above, LS pyramidal neurons display the strongest while CMS pyramidal neurons display the weakest levels of SK channel expression^30^. Interestingly, LS pyramidal neurons also receive the highest levels of serotonergic input while CMS pyramidal neurons received little to no serotonergic input^43^. A recent study has shown that SK channels play a significant role in determining ELL pyramidal neuron responses to envelopes in LS^20^. Indeed, pharmacological inactivation of SK channels strongly increased responses to low frequency envelopes, which is consistent with decreases in adaptation strength^20^. It is thus very likely that increases in serotonin levels will strongly alter LS ELL pyramidal neuron responses to envelopes by downregulating SK channels. We thus predict that increases in serotonin levels will decrease adaptation in LS pyramidal neurons, thereby increasing their responses to the low frequency components of envelopes and making their responses more similar to those of CMS pyramidal neurons. Further studies are needed to verify this prediction and are beyond the scope of this paper.

### Comparison between coding strategies used by the electrosensory system for processing first vs. second-order stimulus features

Comparison of results obtained when considering either the EOD AM or envelope of electrosensory stimuli have revealed that the electrosensory system uses different strategies for each stimulus attribute. These differences start at the level of the sensory periphery. Indeed, electroreceptor afferents display pronounced adaptation in response to step changes in EOD amplitude^48^, thereby performing high-pass filtering of these stimuli^49^,^50^. This adaptation has important functional roles in enabling strong responses to natural electrocommunication stimuli occurring on top of a background^51^,^52^ and possibly optimizing information transmission based on temporal decorrelation of natural AM stimuli^10^. Furthermore, power law adaptation in afferents is necessary in order for the firing rate response to looming objects to be independent of object velocity^53^. Such adaptation however causes ambiguity since a given object, despite giving rise to the same change in EOD amplitude, actually gives rise to different firing rates in a given afferent depending on whether it is looming or receding^54^. The situation is quite different when instead considering envelopes as a recent study has shown that afferent sensitivity to envelopes is actually independent of temporal frequency^18^, implying that peripheral afferents do not display adaptation to this stimulus attribute. Further, correlations between afferents can faithfully track the envelope’s detailed time course^19^. These results imply that afferents can faithfully and unambiguously code for envelopes, which is not the case for AMs as mentioned above.

Differences in coding strategies for AMs and envelopes are also found at the level of the ELL. Indeed, previous studies have found strong differences between the tuning curves of ELL pyramidal neurons to AMs across the CMS, CLS, and LS maps. Indeed, CMS, CLS, and LS neurons tend to be tuned to low, medium, and high temporal frequencies, respectively^55^,^56^. These differences appear to be largely intrinsic in origin and likely involve SK channels and other calcium-dependent mechanisms^32^,^56^. Importantly, differences in levels of expression of SK2 channels between ON and OFF-cells in the CLS and LS maps correlate well with differential adaptation properties, thereby potentially explaining why ON-cells display more phasic responses to AMs than their OFF-type counterparts^32^. In general, ON and OFF-type pyramidal neurons tend to respond in opposite fashion to AM stimuli^20^,^35^,^38^,^57^,^58^,^59^. A recent study has shown synergy between ON and OFF-type cells as the ambiguity in terms of distinguishing between looming and receding motion introduced by adaptation in peripheral afferents is resolved at the level of the ELL by having ON and OFF-type cells respond similarly to looming and receding motion, respectively^54^. The situation is, however, quite different when envelopes are instead considered. Indeed, while previous studies have shown that ELL pyramidal neurons can respond to envelopes^14^,^16^, their tuning has only been reported recently^20^. Interestingly, as mentioned above, no differences were seen between the responses of ON and OFF-type pyramidal neurons to envelopes, which contrasts with their opposite responses to AMs. Scale invariant adaptation in LS cells to envelopes enables optimal encoding of natural envelope stimuli through temporal decorrelation at the single neuron level^20^. Specifically, the responses of ELL LS pyramidal neurons to natural envelope stimuli are then independent of temporal frequency, which is similar in concept to previous results showing that power law adaptation in peripheral afferents makes their responses to looming objects independent of velocity^53^.

While studies including this one have concentrated on how single ELL pyramidal neurons respond to envelopes, it is clear that population coding of envelopes must be considered. This is especially important as ELL pyramidal neurons display correlations in their responses to electrosensory stimuli that are a function of receptive field overlap as well as stimulus statistics^36^,^37^,^60^. While the traditional point of view is that correlated activity is detrimental to information transmission^61^, theoretical studies have shown that correlations can actually benefit information transmission^62^, as recently shown experimentally^63^. While experiments have shown that correlations in CLS and LS ELL pyramidal neurons are strong^36^,^37^,^60^, correlations in CMS have not been measured to this day in *A. leptorhynchus*. The smaller receptive field sizes of CMS neurons, and the consequential lesser degree of receptive field overlap^64^, would suggest that they are weaker in this map. However, it is important to note here that ELL pyramidal neurons are organized in columns each consisting of six cells that receive nearly identical input from peripheral afferents^64^. It is thus likely that, even within CMS, ELL pyramidal neurons within a given column will display significant correlations. Further studies are needed to understand population coding of envelopes by ELL pyramidal neurons and how adaptation in LS, which mediates temporal decorrelation at the single neuron level^20^, affects such coding.

### Parallel coding of behaviorally relevant stimulus features by multiple maps of the body surface

Parallel processing of sensory information is a common strategy used across modalities including auditory^65^,^66^,^67^, visual^68^,^69^,^70^, and electrosensory^7^,^71^,^72^ in order to code for different stimulus attributes. In particular, in the lateral geniculate nucleus, magnocellular cells display strong adaptation to contrast whereas parvocellular cells instead display weak or nonexistent adaptation^73^. Moreover, many systems have multiple representations of the same sensory space by different neural populations, thereby allowing each representation to focus on a subset of behaviorally relevant stimuli^74^,^75^. While previous studies have shown that pyramidal neurons within different maps of the ELL were tuned to different frequency ranges of first order attributes of electrosensory stimuli and displayed differential degrees of adaptation to these attributes^32^,^55^,^56^, which is in agreement with the hypothesis that these maps code distinct features of electrosensory stimuli. Our results showing that pyramidal neurons within different maps display differential degrees of adaptation to the second order attributes of electrosensory stimuli therefore suggest that parallel coding of these attributes is also achieved by these maps.

As mentioned above, further studies are needed in order to determine how pyramidal neurons within each map are tuned to second order attributes of electrosensory stimuli and are beyond the scope of this paper.

Alternatively, it is possible that the weak adaptation displayed by CMS pyramidal neurons enable them to faithfully encode the second order attributes of electrosensory stimuli while the stronger adaptation displayed by LS neurons instead enables them to perform temporal decorrelation^76^. Previous theoretical arguments suggest that efficient coding of natural stimuli through temporal decorrelation should be complemented by a faithful representation of such stimuli^77^. LS pyramidal neurons then efficiently encode natural second order stimuli through temporal decorrelation as shown recently^20^ while CMS pyramidal neurons instead provide a faithful representation of these stimuli. Input from both channels would be necessary for decoding in downstream areas such as TS.

## Materials and Methods

### Animals

All procedures have been described previously^16^,^27^,^37^,^44^,^52^,^78^ and were approved by the McGill University Animal Care Committee. All experiments were performed in accordance with the guidelines and regulations of the Canadian Council on Animal Care. Weakly electric fish *Apteronotus leptorhynchus* of either sex were obtained from tropical fish suppliers and acclimated to laboratory conditions as per published guidelines^79^.

### Surgery and recordings

Animals were injected intramuscularly with Tubocurarine chloride pentahydrate (Sigma, 1 μg/g body weight), and then respirated with water flowing over the gills at a rate of ∼10 mL/min. The fish was kept submerged in water except for the top of its head. Local anesthetic (2% lidocaine) was applied prior to removing ∼6 mm^2^ of the skin above the skull and gluing a metal post to the skull surface to ensure stability. A ∼2 mm^2^ hole through the skull was then drilled over the eminentia granularis posterior over the ELL. We used metal-filled micropipettes^80^ to obtain extracellular recordings from ELL pyramidal neurons within the lateral (LS) and centromedial (CMS) segments. As before, we used surface landmarks as well as the depth of recording in order to differentiate between both segments^56^,^81^. The resulting signal was amplified (Model 1000 amplifier, A-M systems) and sampled at 10 KHz prior to being stored on the computer using a CED Power1401 and Spike2 software.

### Stimulus

Our stimuli consisted of amplitude modulations (AMs) of the animal’s own EOD that persists after curare injection. It is important to note here that the EOD is a carrier signal and that the relevant stimulus for ELL pyramidal neurons is the EOD AM. The animal’s EOD was recorded with chloridized silver wires placed near the head and tail. The EOD’s zero-crossings with a positive slope were used to trigger a function generator (Agilent 33220A) in order to create a train of sinusoidal cycles at a frequency slightly (40 Hz) higher than the EOD frequency. This train was then multiplied with the EOD AM stimulus (MT3 multiplier, Tucker Davis Technologies), attenuated (LAT45 attenuator, Leader Electronics), and then isolated from ground (A395 linear stimulus isolator, World Precision Instruments), prior to being delivered to the experimental tank via two chloridized silver wires located ∼20 cm on each side of the fish. Stimulus intensity was monitored by a small dipole located ∼2 mm from the skin surface and placed at the animal’s midpoint with respect to both the rostro-caudal and dorso-ventral axes. Stimulus contrast (i.e the ratio of the AM to the baseline EOD amplitude) was about 15% as in previous studies^18^,^19^,^36^,^37^,^44^.

AM stimuli consisted of 4Hz sinusoids as well as band-pass (5–15 Hz and 60–80 Hz, fourth-order Butterworth) filtered white noise (i.e. first order) whose amplitude (i.e. the envelope or second order) was modulated in a stepwise fashion at frequencies 0.05, 0.1, 0.25, 0.5, 1, 2, and 4 Hz for 5–15 Hz and 0.05, 0.1, 0.25, 0.5, 1, 2, 4, 8, and 16 Hz for 60–80 Hz. The 5–15 Hz and 60–80 Hz first order modulations mimic signals that occur during encounters between same and opposite-sex conspecifics, respectively. Changes in the distance and orientation between two or more conspecifics will give rise to a time-varying envelope signal under natural conditions^9^,^10^,^11^,^12^. Each stimulus was presented for a sufficiently long duration such that responses could be averaged for at least 15 cycles (for low frequencies) and up to 180 cycles (for high frequencies). All stimuli were generated in Matlab (Mathworks).

### Data analysis

Data analysis was also performed in Matlab. We first high-pass filtered (eighth-order Butterworth, 300 Hz cutoff) the digitized extracellular recording and action potential times were obtained as the times at which the signal crossed an appropriately-chosen threshold. These were then used to make a binary representation of the spike train at 10 kHz by setting the value of a given bin to 1 if an action potential occurred within it and 0 otherwise. Since no significant differences were observed when comparing responses to 5–15 Hz and 60–80 Hz noises, data were pooled.

Pyramidal neurons were first classified as either ON or OFF-type based on whether they responded to the upstrokes or downstrokes of a 4 Hz sinusoidal stimulus^6^. We then constructed peri-stimulus time histograms (PSTHs) by averaging over each step onset and offset and typically used 50 bins for a given step duration. The tendency to display spike frequency adaptation (i.e. the adaptation strength) was quantified as the change in firing rate in response to the step onset for a 0.1 Hz frequency (i.e. the difference between the peak firing rate and the firing rate just prior to the step offset).

#### Time course of spike frequency adaptation

The time course of spike frequency adaptation was characterized by first estimating the steady state firing rate from PSTHs generated using evenly spaced bins. The steady state firing rate was then subtracted from PSTHs generated using logarithmically spaced bins prior to normalizing by, and aligned on, the maximum value. The normalized PSTHs were then fitted using Matlab’s “nlinfit” function using either exponential or power law curves:
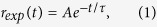

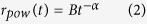
where *r*_*exp*_(*t*) and *r*_*pow*_(*t*) are now the normalized respective firing rates for the exponential and power law fits, *A*, *B* are constants, *τ* is the exponential time constant in seconds, and *α* is the power law exponent. We note that normalization does not alter the values of either *τ* or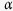.

#### Receiver operating characteristic analysis

We quantified the ability of an ideal observer to distinguish between low and high envelope values using receiver-operator characteristic (ROC) analysis of single neuron activity. We computed the probability distributions of firing rate for a single cell over the course of a 0.1 Hz switch by separating the time course into 500 ms bins, computing the firing rate during each bin over each repetition, and creating a histogram of those firing rates. To compute the ability to discriminate an input envelope, we compared the firing rate distributions just before and at several times after the step onset. The probabilities of correct detection (PD) and false alarm (PFA) were computed by integrating the distributions up to a threshold and the ROC curve was obtained by plotting PD as a function of PFA while systematically moving the threshold^21^. We quantified discriminability by computing the area under the ROC curve (AuC). A value of 1 indicates perfect discrimination while a value of 0.5 indicates chance level.

To compute the population-averaged discriminabilities of LS and CMS pyramidal neurons, we normalized the firing rate probability distributions for each cell with respect to the reference distribution taken from just before the step onset, setting the mean of that distribution to zero and scaling all other distributions by the standard deviation of the reference. This normalization allows us to compare firing rate histograms between cells. We then computed the ROC curve of each cell separately from these distributions. To compute the average ROC curve, we first rotated all points of the curve clockwise by 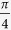 radians. We binned the interval 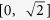 into 7 bins, set the “centre” of each bin as the average abscissa value of the points contained in the bin, the mean of the ROC curve as the mean of the ordinate values in that bin, and the 95% confidence intervals of the ROC curve as the 95% confidence intervals of those same ordinate values. These points are then rotated counter-clockwise 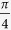 radians back to the ROC-style orientation.

## Additional Information

**How to cite this article**: Zhang, Z. D. and Chacron, M. J. Adaptation to second order stimulus features by electrosensory neurons causes ambiguity. *Sci. Rep.* **6**, 28716; doi: 10.1038/srep28716 (2016).

## References

[b1] Wark B., Lundstrom B. N. & Fairhall A. Sensory adaptation. Current Opinion in Neurobiology 17, 423–429 (2007). 17714934 10.1016/j.conb.2007.07.001 PMC2084204

[b2] Simoncelli E. P. & Olshausen B. A. Natural image statistics and neural representation. Annual Review of Neuroscience 24, 1193–1216 (2001). 10.1146/annurev.neuro.24.1.1193 11520932

[b3] Laughlin S. A simple coding procedure enhances a neuron’s information capacity. Z Naturforsch C 36, 910–912 (1981). 7303823

[b4] Fairhall A. L., Lewen G. D., Bialek W. & de Ruyter van Steveninck R. R. Efficiency and ambiguity in an adaptive neural code. Nature 412, 787–792 (2001). 11518957 10.1038/35090500

[b5] Ollerenshaw D. R., Zheng H. J. V., Millard D. C., Wang Q. & Stanley G. B. The Adaptive Trade-Off between Detection and Discrimination in Cortical Representations and Behavior. Neuron 81, 1152–1164 (2014). 24607233 10.1016/j.neuron.2014.01.025 PMC4026261

[b6] Chacron M. J., Longtin A. & Maler L. Efficient computation via sparse coding in electrosensory neural networks. Curr Opin Neurobiol 21, 752–760 (2011). 21683574 10.1016/j.conb.2011.05.016 PMC4529320

[b7] Bell C. & Maler L. Central neuroanatomy of electrosensory systems in fish. In Electroreception (ed. Bullock T. H., Hopkins C. D., Popper A. N. & Fay R. R.) 68–111 (Springer, New York, 2005).

[b8] Krahe R. & Maler L. Neural maps in the electrosensory system of weakly electric fish. Curr Opin Neurobiol 24, 13–21 (2014). 24492073 10.1016/j.conb.2013.08.013

[b9] Yu N., Hupe G. J., Garfinkle C., Lewis J. E. & Longtin A. Coding Conspecific Identity and Motion in the Electric Sense. PLoS Comp Biol 8, e1002564 (2012). 10.1371/journal.pcbi.1002564 PMC3395610 22807662

[b10] Fotowat H., Harrison R. R. & Krahe R. Statistics of the Electrosensory Input in the Freely Swimming Weakly Electric Fish Apteronotus leptorhynchus. J Neurosci 33, 13758–13772 (2013). 23966697 10.1523/JNEUROSCI.0998-13.2013 PMC6618647

[b11] Metzen M. G. & Chacron M. J. Weakly electric fish display behavioral responses to envelopes naturally occurring during movement: implications for neural processing. J Exp Biol 217, 1381–1391 (2014). 24363423 10.1242/jeb.098574 PMC4529328

[b12] Stamper S. A., Fortune E. S. & Chacron M. J. Perception and coding of envelopes in weakly electric fishes. J Exp Biol 216, 2393–2402 (2013). 23761464 10.1242/jeb.082321 PMC4529321

[b13] Marsat G., Longtin A. & Maler L. Cellular and circuit properties supporting different sensory coding strategies in electric fish and other systems. Curr Opin Neurobiol 22, 686–692 (2012). 22326255 10.1016/j.conb.2012.01.009

[b14] Middleton J. W., Longtin A., Benda J. & Maler L. The cellular basis for parallel neural transmission of a high-frequency stimulus and its low-frequency envelope. PNAS 103, 14596–14601 (2006). 16983081 10.1073/pnas.0604103103 PMC1600005

[b15] Savard M., Krahe R. & Chacron M. J. Neural heterogeneities influence envelope and temporal coding at the sensory periphery. Neuroscience 172, 270–284 (2011). 21035523 10.1016/j.neuroscience.2010.10.061 PMC4529325

[b16] McGillivray P., Vonderschen K., Fortune E. S. & Chacron M. J. Parallel coding of first- and second-order stimulus attributes by midbrain electrosensory neurons. J Neurosci 32, 5510–5524 (2012). 22514313 10.1523/JNEUROSCI.0478-12.2012 PMC4535166

[b17] Metzen M. G., Avila-Akerberg O. & Chacron M. J. Coding stimulus amplitude by correlated neural activity. Physical review. E, Statistical, nonlinear, and soft matter physics 91, 042717 (2015). 10.1103/PhysRevE.91.042717 PMC4461379 25974537

[b18] Metzen M. G. & Chacron M. J. Neural heterogeneities determine response characteristics to second-, but not first-order stimulus features. J Neurosci 35, 3124–3138 (2015). 25698748 10.1523/JNEUROSCI.3946-14.2015 PMC4529327

[b19] Metzen M. G. . Coding of envelopes by correlated but not single-neuron activity requires neural variability. PNAS 112, 4791–4796 (2015). 25825717 10.1073/pnas.1418224112 PMC4403158

[b20] Huang C. G., Zhang Z. D. & Chacron M. J. Temporal decorrelation by SK channels enables efficient neural coding and perception of natural stimuli. Nature communications 7, 11353 (2016). 10.1038/ncomms11353 PMC4837484 27088670

[b21] Green D. & Swets J. Signal Detection Theory and Psychophysics (John Wiley & Sons, New York, 1966).

[b22] Lundstrom B. N., Fairhall A. L. & Maravall M. Multiple timescale encoding of slowly varying whisker stimulus envelope in cortical and thalamic neurons *in vivo*. J Neurosci 30, 5071–5077 (2010). 20371827 10.1523/JNEUROSCI.2193-09.2010 PMC6632796

[b23] Hildebrandt K. J., Ronacher B., Hennig R. M. & Benda J. A neural mechanism for time-window separation resolves ambiguity of adaptive coding. PLoS Biol 13, e1002096 (2015). 25761097 10.1371/journal.pbio.1002096 PMC4356587

[b24] Chacron M. J. & Fortune E. S. Subthreshold membrane conductances enhance directional selectivity in vertebrate sensory neurons. J Neurophysiol 104, 449–462 (2010). 20445028 10.1152/jn.01113.2009 PMC4850070

[b25] Chacron M. J., Toporikova N. & Fortune E. S. Differences in the time course of short-term depression across receptive fields are correlated with directional selectivity in electrosensory neurons. J Neurophysiol 102, 3270–3279 (2009). 19793877 10.1152/jn.00645.2009 PMC4850067

[b26] Khosravi-Hashemi N., Fortune E. S. & Chacron M. J. Coding movement direction by burst firing in electrosensory neurons. J Neurophysiol 106, 1954–1968 (2011). 21775723 10.1152/jn.00116.2011 PMC4850071

[b27] Vonderschen K. & Chacron M. J. Sparse and dense coding of natural stimuli by distinct midbrain neuron subpopulations in weakly electric fish. J Neurophysiol 106, 3102–3118 (2011). 21940609 10.1152/jn.00588.2011 PMC4535167

[b28] Sproule M. K. J., Metzen M. G. & Chacron M. J. Parallel sparse and dense information coding streams in the electrosensory midbrain. Neurosci Lett 607, 1–6 (2015). 26375927 10.1016/j.neulet.2015.09.014 PMC4633296

[b29] Aumentado-Armstrong T., Metzen M. G., Sproule M. K. J. & Chacron M. J. Electrosensory Midbrain Neurons Display Feature Invariant Responses to Natural Communication Stimuli. PLoS Comput Biol 11, e1004430 (2015). 26474395 10.1371/journal.pcbi.1004430 PMC4608831

[b30] Ellis L. D., Maler L. & Dunn R. J. Differential distribution of SK channel subtypes in the brain of the weakly electric fish Apteronotus leptorhynchus. Journal of Comparative Neurology 507, 1964–1978 (2008). 18273887 10.1002/cne.21597

[b31] Ellis L. D. . SK channels provide a novel mechanism for the control of frequency tuning in electrosensory neurons. J Neurosci 27, 9491–9502 (2007). 17728462 10.1523/JNEUROSCI.1106-07.2007 PMC6673139

[b32] Mehaffey W. H., Maler L. & Turner R. W. Intrinsic frequency tuning in ELL pyramidal neurons varies across electrosensory maps. J Neurophysiol 99, 2641–2655 (2008). 18367702 10.1152/jn.00028.2008

[b33] Maler L. The posterior lateral line lobe of certain gymnotiform fish. Quantitative light microscopy. Journal of Comparative Neurology 183, 323–363 (1979). 762262 10.1002/cne.901830208

[b34] Maler L., Sas E. K. & Rogers J. The cytology of the posterior lateral line lobe of high frequency weakly electric fish (Gymnotidae): Differentiation and synaptic specificity in a simple cortex. Journal of Comparative Neurology 195, 87–139 (1981). 7204653 10.1002/cne.901950107

[b35] Bastian J., Chacron M. J. & Maler L. Plastic and non-plastic cells perform unique roles in a network capable of adaptive redundancy reduction. Neuron 41, 767–779 (2004). 15003176 10.1016/s0896-6273(04)00071-6

[b36] Chacron M. J. & Bastian J. Population coding by electrosensory neurons. J Neurophysiol 99, 1825–1835 (2008). 18256161 10.1152/jn.01266.2007 PMC4844541

[b37] Simmonds B. & Chacron M. J. Activation of parallel fiber feedback by spatially diffuse stimuli simultaneously reduces signal and noise correlations via independent mechanisms in a cerebellum-like structure. PLoS Comp Biol 11, e1004034 (2015). 10.1371/journal.pcbi.1004034 PMC4287604 25569283

[b38] Chacron M. J., Maler L. & Bastian J. Feedback and Feedforward Control of Frequency Tuning to Naturalistic Stimuli. J Neurosci 25, 5521–5532 (2005). 15944380 10.1523/JNEUROSCI.0445-05.2005 PMC5053810

[b39] Chacron M. J., Doiron B., Maler L., Longtin A. & Bastian J. Non-classical receptive field mediates switch in a sensory neuron’s frequency tuning. Nature 423, 77–81 (2003). 12721628 10.1038/nature01590

[b40] Doiron B., Chacron M. J., Maler L., Longtin A. & Bastian J. Inhibitory feedback required for network oscillatory responses to communication but not prey stimuli. Nature 421, 539–543 (2003). 12556894 10.1038/nature01360

[b41] Mejias J. F., Marsat G., Bol K., Maler L. & Longtin A. Learning contrast-invariant cancellation of redundant signals in neural systems. PLoS Comput Biol 9, e1003180 (2013). 24068898 10.1371/journal.pcbi.1003180 PMC3772051

[b42] Bastian J. Gain control in the electrosensory system mediated by descending inputs to the electrosensory lateral line lobe. J Neurosci 6, 553–562. (1986). 3950710 10.1523/JNEUROSCI.06-02-00553.1986 PMC6568544

[b43] Deemyad T., Maler L. & Chacron M. J. Inhibition of SK and M channel-mediated currents by 5-HT enables parallel processing by bursts and isolated spikes. J Neurophysiol 105, 1276–1294 (2011). 21209357 10.1152/jn.00792.2010 PMC4850069

[b44] Deemyad T., Metzen M. G., Pan Y. & Chacron M. J. Serotonin selectively enhances perception and sensory neural responses to stimuli generated by same-sex conspecifics. PNAS 110, 19609–19614 (2013). 24218585 10.1073/pnas.1314008110 PMC3845146

[b45] Ellis L. D., Krahe R., Bourque C. W., Dunn R. J. & Chacron M. J. Muscarinic receptors control frequency tuning through the downregulation of an A-type potassium current. J Neurophysiol 98, 1526–1537 (2007). 17615127 10.1152/jn.00564.2007 PMC5053812

[b46] Mehaffey W. H., Ellis L. D., Krahe R., Dunn R. J. & Chacron M. J. Ionic and Neuromodulatory Regulation of Burst Discharge Controls Frequency Tuning. J Physiol (Paris) 102, 195–208 (2008). 18992813 10.1016/j.jphysparis.2008.10.019 PMC4529324

[b47] Marquez B. T., Krahe R. & Chacron M. J. Neuromodulation of early electrosensory processing in gymnotiform weakly electric fish. J Exp Biol 216, 2442–2450 (2013). 23761469 10.1242/jeb.082370 PMC4529319

[b48] Xu Z., Payne J. R. & Nelson M. E. Logarithmic time course of sensory adaptation in electrosensory afferent nerve fibers in a weakly electric fish. J Neurophysiol 76, 2020–2032 (1996). 8890311 10.1152/jn.1996.76.3.2020

[b49] Nelson M. E., Xu Z. & Payne J. R. Characterization and modeling of P-type electrosensory afferent responses to amplitude modulations in a wave-type electric fish. Journal of Comparative Physiology A-Sensory Neural & Behavioral Physiology 181, 532–544 (1997). 10.1007/s003590050137 9373958

[b50] Chacron M. J., Maler L. & Bastian J. Electroreceptor Neuron Dynamics Shape Information Transmission. Nature Neuroscience 8, 673–678 (2005). 15806098 10.1038/nn1433 PMC5283878

[b51] Benda J., Longtin A. & Maler L. Spike-frequency adaptation separates transient communication signals from background oscillations. J Neurosci 25, 2312–2321 (2005). 15745957 10.1523/JNEUROSCI.4795-04.2005 PMC6726095

[b52] Metzen M. G., Hofmann V. & Chacron M. J. Neural correlations enable invariant coding and perception of natural stimuli in weakly electric fish. Elife 5, e12993 (2016). 27128376 10.7554/eLife.12993 PMC4851552

[b53] Clarke S. E., Naud R., Longtin A. & Maler L. Speed-invariant encoding of looming object distance requires power law spike rate adaptation. PNAS 110, 13624–13629 (2013). 23898185 10.1073/pnas.1306428110 PMC3746935

[b54] Clarke S. E., Longtin A. & Maler L. A neural code for looming and receding motion is distributed over a population of electrosensory ON and OFF contrast cells. J Neurosci 34, 5583–5594 (2014). 24741048 10.1523/JNEUROSCI.4988-13.2014 PMC6608223

[b55] Shumway C. Multiple electrosensory maps in the medulla of weakly electric Gymnotiform fish. I. Physiological differences. J Neurosci 9, 4388–4399 (1989). 2593005 10.1523/JNEUROSCI.09-12-04388.1989 PMC6569630

[b56] Krahe R., Bastian J. & Chacron M. J. Temporal processing across multiple topographic maps in the electrosensory system. J Neurophysiol 100, 852–867 (2008). 18509073 10.1152/jn.90300.2008 PMC2525725

[b57] Saunders J. & Bastian J. The physiology and morphology of two classes of electrosensory neurons in the weakly electric fish *Apteronotus Leptorhynchus*. Journal of Comparative Physiology A 154, 199–209 (1984).

[b58] Bastian J., Chacron M. J. & Maler L. Receptive field organization determines pyramidal cell stimulus-encoding capability and spatial stimulus selectivity. J Neurosci 22, 4577–4590 (2002). 12040065 10.1523/JNEUROSCI.22-11-04577.2002 PMC6758818

[b59] Chacron M. J. Nonlinear information processing in a model sensory system. J Neurophysiol 95, 2933–2946 (2006). 16495358 10.1152/jn.01296.2005 PMC5053817

[b60] Litwin-Kumar A., Chacron M. J. & Doiron B. The spatial structure of stimuli shapes the timescale of correlations in population spiking activity. PLoS Comput Biol 8, e1002667 (2012). 23028274 10.1371/journal.pcbi.1002667 PMC3441501

[b61] Zohary E., Shadlen M. N. & Newsome W. T. Correlated neuronal discharge rate and its implications for psychophysical performance. Nature 370, 140–143 (1994). 8022482 10.1038/370140a0

[b62] Averbeck B. B., Latham P. E. & Pouget A. Neural correlations, population coding and computation. Nat Rev Neurosci 7, 358–366 (2006). 16760916 10.1038/nrn1888

[b63] Franke F. . Structures of Neural Correlation and How They Favor Coding. Neuron 89, 409–422 (2016). 26796692 10.1016/j.neuron.2015.12.037 PMC5424879

[b64] Maler L. Receptive field organization across multiple electrosensory maps. I. Columnar organization and estimation of receptive field size. Journal of Comparative Neurology 516, 376–393 (2009). 19655387 10.1002/cne.22124

[b65] Takahashi T., Moiseff A. & Konishi M. Time and intensity cues are processed independently in the auditory system of the owl. J Neurosci 4, 1781–1786 (1984). 6737040 10.1523/JNEUROSCI.04-07-01781.1984 PMC6564890

[b66] Oertel D. The role of timing in the brain stem auditory nuclei of vertebrates. Annual review of physiology 61, 497–519 (1999). 10.1146/annurev.physiol.61.1.497 10099699

[b67] Gelfand S. Hearing: An Introduction to Psychological and Physiological Acoustics (Informa Healthcare, Colchester, 2004).

[b68] Marr D. Vision (Freeman, New York, 1982).

[b69] Livingstone M. S. & Hubel D. H. Psychophysical evidence for separate channels for the perception of form, color, movement, and depth. J Neurosci 7, 3416–3468 (1987). 3316524 10.1523/JNEUROSCI.07-11-03416.1987 PMC6569044

[b70] Merigan W. H. & Maunsell J. H. How parallel are the primate visual pathways? Annual Review of Neuroscience 16, 369–402 (1993). 10.1146/annurev.ne.16.030193.002101 8460898

[b71] Carr C. E. & Maler L. Electroreception in gymnotiform fish. Central anatomy and physiology. In Electroreception (ed. Bullock T. H. & Heiligenberg W.) 319–373 (Wiley, New York, 1986).

[b72] Kawasaki M. Central neuroanatomy of electrosensory systems in fish. In Electroreception (ed. Bullock T. H., Hopkins C. D., Popper A. N. & Fay R. R.) 154–194 (Springer, New York, 2005).

[b73] Solomon S. G., Peirce J. W., Dhruv N. T. & Lennie P. Profound contrast adaptation early in the visual pathway. Neuron 42, 155–162 (2004). 15066272 10.1016/s0896-6273(04)00178-3

[b74] Wassle H. Parallel processing in the mammalian retina. Nat Rev Neurosci 5, 747–757 (2004). 15378035 10.1038/nrn1497

[b75] MacLeod K. M. & Carr C. E. Beyond timing in the auditory brainstem: intensity coding in the avian cochlear nucleus angularis. Prog Brain Res 165, 123–133 (2007). 17925243 10.1016/S0079-6123(06)65008-5 PMC3286339

[b76] Pozzorini C., Naud R., Mensi S. & Gerstner W. Temporal whitening by power-law adaptation in neocortical neurons. Nat Neurosci 16, 942–948 (2013). 23749146 10.1038/nn.3431

[b77] Barlow H. Redundancy reduction revisited. Network 12, 241–253 (2001). 11563528

[b78] Toporikova N. & Chacron M. J. Dendritic SK channels gate information processing *in vivo* by regulating an intrinsic bursting mechanism seen *in vitro*. J Neurophysiol 102, 2273–2287 (2009). 19675292 10.1152/jn.00282.2009 PMC4850068

[b79] Hitschfeld E. M., Stamper S. A., Vonderschen K., Fortune E. S. & Chacron M. J. Effects of restraint and immobilization on electrosensory behaviors of weakly electric fish. ILAR J 50, 361–372 (2009). 19949252 10.1093/ilar.50.4.361 PMC4844542

[b80] Frank K. & Becker M. C. Microelectrodes for recording and stimulation. In Physical Techniques in Biological Research (ed. Nastuk W. L.) 23–84 (Academic, New York, 1964).

[b81] Khosravi-Hashemi N. & Chacron M. J. Motion processing across multiple topographic maps in the electrosensory system. Physiol Rep 2, e00253 (2014). 24760508 10.1002/phy2.253 PMC4002234

